# Heterogeneity Diffusion Imaging of gliomas: Initial experience and validation

**DOI:** 10.1371/journal.pone.0225093

**Published:** 2019-11-14

**Authors:** Qing Wang, Gloria J. Guzmán Pérez-Carrillo, Maria Rosana Ponisio, Pamela LaMontagne, Sonika Dahiya, Daniel S. Marcus, Mikhail Milchenko, Joshua Shimony, Jingxia Liu, Gengsheng Chen, Amber Salter, Parinaz Massoumzadeh, Michelle M. Miller-Thomas, Keith M. Rich, Jonathan McConathy, Tammie L. S. Benzinger, Yong Wang

**Affiliations:** 1 Department of Radiology, Washington University in St. Louis, St. Louis, Missouri, United States of America; 2 Department of Medical Imaging, Neuroradiology Section, University of Arizona, Tucson, Arizona, United States of America; 3 Department of Pathology and Immunology, Washington University in St. Louis, St. Louis, Missouri, United States of America; 4 Department of Surgery, Washington University in St. Louis, St. Louis, Missouri, United States of America; 5 Department of Biostatistics, Washington University in St. Louis, St. Louis, Missouri, United States of America; 6 Department of Neurosurgery, Washington University in St. Louis, St. Louis, Missouri, United States of America; 7 Department of Radiology, Division of Molecular Imaging and Therapeutics, University of Alabama at Birmingham, Birmingham, Alabama, United States of America; 8 Department of Obstetrics and Gynecology, Washington University in St. Louis, St. Louis, Missouri, United States of America; University of Central Florida (UCF), UNITED STATES

## Abstract

**Objectives:**

Primary brain tumors are composed of tumor cells, neural/glial tissues, edema, and vasculature tissue. Conventional MRI has a limited ability to evaluate heterogeneous tumor pathologies. We developed a novel diffusion MRI-based method—Heterogeneity Diffusion Imaging (HDI)—to simultaneously detect and characterize multiple tumor pathologies and capillary blood perfusion using a single diffusion MRI scan.

**Methods:**

Seven adult patients with primary brain tumors underwent standard-of-care MRI protocols and HDI protocol before planned surgical resection and/or stereotactic biopsy. Twelve tumor sampling sites were identified using a neuronavigational system and recorded for imaging data quantification. Metrics from both protocols were compared between World Health Organization (WHO) II and III tumor groups. Cerebral blood volume (CBV) derived from dynamic susceptibility contrast (DSC) perfusion imaging was also compared with the HDI-derived perfusion fraction.

**Results:**

The conventional apparent diffusion coefficient did not identify differences between WHO II and III tumor groups. HDI-derived slow hindered diffusion fraction was significantly elevated in the WHO III group as compared with the WHO II group. There was a non-significantly increasing trend of HDI-derived tumor cellularity fraction in the WHO III group, and both HDI-derived perfusion fraction and DSC-derived CBV were found to be significantly higher in the WHO III group. Both HDI-derived perfusion fraction and slow hindered diffusion fraction strongly correlated with DSC-derived CBV. Neither HDI-derived cellularity fraction nor HDI-derived fast hindered diffusion fraction correlated with DSC-derived CBV.

**Conclusions:**

Conventional apparent diffusion coefficient, which measures averaged pathology properties of brain tumors, has compromised accuracy and specificity. HDI holds great promise to accurately separate and quantify the tumor cell fraction, the tumor cell packing density, edema, and capillary blood perfusion, thereby leading to an improved microenvironment characterization of primary brain tumors. Larger studies will further establish HDI’s clinical value and use for facilitating biopsy planning, treatment evaluation, and noninvasive tumor grading.

## Introduction

Gliomas account for the majority of primary brain tumors in adults; they represent 26.5% of primary brain tumors and 80.7% of malignant brain tumors [1]. Typically, malignant gliomas contain heterogeneous pathologies that reflect regional diversity in tumor cell proliferation [2], immune infiltration, tumor vessel density, necrosis, and cystic degeneration. This heterogeneity makes clinical diagnosis and management very challenging.

Current standard-of-care imaging for newly diagnosed patients with brain tumors includes anatomical magnetic resonance imaging (MRI) with and without contrast to identify general characteristic features of the tumor, including its location, size, and extent. However, anatomical MRI techniques alone are limited for the evaluation of tumor heterogeneity, especially in tumors that demonstrate little or no enhancement. Advanced MRI techniques such as diffusion MRI [3, 4], MR spectroscopy [5, 6] and MR perfusion [7] provide more pathophysiologic information, and they have demonstrated the potential to characterize tumor types and to differentiate recurrent tumor from pseudo progression. Among these advanced techniques, diffusion MRI holds a unique position due to its sensitivity when probing the microenvironment of biological tissues at a cellular level. Clinically, apparent diffusion coefficient (ADC) maps have been used to grade primary brain tumors [8, 9], to define tumor cellularity [10], and to assess brain tumor response to therapy [11, 12]. However, conventional ADC measures average the diffusivity of multiple pathologies coexisting within each tumor voxel, which significantly limits the accuracy and specificity of this method for characterizing neoplastic pathologies.

Perfusion MRI provides important diagnostic information about microvascularity within brain tumors, and it can be used to differentiate tumor types and tumor grades [7, 13]. Quantitative and physiologic information provided by diffusion and perfusion MRI are complimentary, and the integration of these two techniques could significantly improve diagnostic confidence [14]. However, imaging properties examined by different imaging techniques may suffer mislocalization [15], which could increase registration errors and decrease diagnostic accuracy. The accurate and simultaneous imaging and quantification of tumor pathological heterogeneity and vascularity—in one session—will be highly favored to reduce registration error, thereby improving clinical diagnosis, treatment, and management.

In this article, we present and examine a novel diffusion MRI technique called *Heterogeneity Diffusion Imaging* (HDI), which is capable of characterizing heterogeneous tumor composition and microvascularity simultaneously from a single clinical diffusion MRI scan. HDI employs a multiple diffusion compartment model, which was developed based on diffusion basis spectrum imaging (DBSI) [16, 17]. By modeling the confounding effects of anatomical complexities and neuropathologies, this approach is able to disentangle the heterogeneous pathological components that are mixed in one imaging voxel [16–20]. In the current study, HDI was employed to quantify tumor cellularity, slow versus fast hindered diffusion fraction, and perfusion effect in each tumor voxel. We hypothesized that HDI would be more sensitive for the characterization of gliomas’ heterogeneous microenvironments as compared with conventional ADC.

## Materials and methods

### Patient selection

This prospective single-center pilot study was approved by the Washington University in St. Louis Institutional Review Board. Seven adults with primary brain tumors were recruited from the Washington University School of Medicine between 2015 and 2016. They underwent the standard-of-care imaging protocol and HDI diffusion MRI before planned standard-of-care surgical resection and/or stereotactic biopsy. The study was carried out in accordance with the guidelines of the institutional review board of the Washington University Human Research Protection Office. Written informed consent was obtained from all participants. The age range, patient tumor type, and tumor grade are summarized in Table 1. There were four female and three male subjects in this study. Areas of tumor sampling were identified intraoperatively using the Stealth neuronavigational system (Medtronic, Minneapolis, MN, USA); the location and spatial coordinates of tissue sampling sites were recorded to align with HDI results. Each subject had at least one recorded tissue sampling site. Twelve tumor samples were prepared for this study. The entire HDI analysis was performed blinded to all other data (i.e., clinical data and information about patient surgeries and outcomes).

**Table 1:** Characteristics of patients and tumor grades detected by biopsy.

Patient No.	Age Range (years)	Tumor Pathology	Tumor Grade	IDH Mutant
**S1**	**40s**	**Oligodendroglioma**	**WHO III**	**Yes**
**S3**	**60s**	**Oligodendroglioma**	**WHO II**	**Yes**
**S4**	**30s**	**Oligodendroglioma**	**WHO II**	**Yes**
**S6**	**30s**	**Anaplastic astrocytoma**	**WHO III**	**Yes**
**S7**	**30s**	**Astrocytoma**	**WHO II**	**Yes**
**S8**	**30s**	**Oligodendroglioma**	**WHO II**	**Yes**
**S9**	**40s**	**Oligodendroglioma**	**WHO III**	**Yes**

IDH, Isocitrate dehydrogenase; WHO, World Health Organization.

### MRI data acquisition

MRI scans were performed on a 3-Tesla positron emission tomography (PET)/MRI system, the Siemens Biograph mMR (Siemens Health Care, Erlangen, Germany). MR images were acquired via the Comprehensive Neuro-Oncology Data Repository (CONDR) imaging protocol used for brain tumors [21]. The protocol included pre-gadolinium and post-gadolinium T1-weighted (T_1_W) imaging (TR = 18 ms, TE = 4.38 ms, in-plane resolution = 1.0 × 1.0 mm^2^, slice thickness = 1.0 mm); magnetization-prepared rapidly acquired gradient echo (TR = 2300 ms, TE = 2.95 ms, TI = 900 ms, in-plane resolution = 1.0 × 1.0 mm^2^, slice thickness = 1.0 mm); dynamic susceptibility contrast (DSC) perfusion-weighted imaging (TR = 1930 ms, TE = 36 ms, in-plane resolution = 2.2 × 2.2 mm^2^, slice thickness = 5.0 mm); T2-weighted (T_2_W) fluid-attenuated inversion recovery (FLAIR) (TR = 8500 ms, TE = 133 ms, flip angle = 130 degrees, in-plane resolution = 0.9 × 0.9 mm^2^, slice thickness = 5.0 mm); and standard diffusion tensor imaging (DTI) (TR = 9000 ms, TE = 91 ms, in-plane resolution = 2.2 × 2.2 mm^2^, slice thickness = 3.0 mm). Clinical DTI scans were acquired using a 12-direction gradient scheme with a maximum *b* value of 1000 s/mm^2^.

We also designed and incorporated a diffusion scheme for HDI data acquisition that was used in addition to the clinical DTI scan. The new diffusion protocol included 74 diffusion directions distributed uniformly in the three-dimensional space, with 74 different *b* values; it was administered across four separate sessions to improve patient tolerance. Each diffusion gradient had a unique *b* value, and all of the *b* values were uniformly distributed between 0 and 2000 s/mm^2^. The maximum *b* value for each session was 2000 s/mm^2^. The imaging parameters were as follows: TR = 9500 ms; TE = 93 ms; in-plane resolution = 2.0 × 2.0 mm^2^; and slice thickness = 2.0 mm.

### MRI image processing

#### MRI preprocessing

For each individual subject, all MRI sequences were coregistered to a target post-contrast T_1_W image using the Multimodal Glioma Analysis (MGA) pipeline [22]. Each subject’s T_1_W image was registered to a T_1_W atlas template image, and other T_1_W and T_2_W sequences were coregistered with the subject’s T_1_W target image. T_1_W → T_1_W registration used the maximization of spatial correlation [23], whereas cross-modal registration (e.g., T_2_W → T_1_W) used the alignment of intensity gradients [24]. Perfusion and diffusion parameter maps were transformed to the T_1_W target space using a transformation matrix obtained from coregistering respective sequences. The coregistration quality was verified using built-in MGA quality control metrics.

#### Diffusion and perfusion processing

After the acquisition and registration steps were completed, each subject’s raw diffusion and perfusion data were processed. The MGA pipeline was used for diffusion and perfusion processing. MGA precedes perfusion modeling by correcting signal intensity across slices and registering all frames to a middle time frame. Perfusion modeling is initialized by the automatic estimation of a local arterial input function and the selection of a convolution/deconvolution method. The arterial input function is defined using the Bayesian tissue model [25]. Diffusion data were processed based on the standard DTI model [26]. With the use of these methods, MGA computed the cerebral blood volume (CBV), mean transit time, and cerebral blood flow maps for the DSC scans. For diffusion-weighted scans, the ADC was computed.

#### HDI processing

Diffusion data acquired with the use of our new diffusion protocol were analyzed using the HDI method. HDI was developed based on the multiple tensor formulations used in the DBSI model [16, 17]. Briefly, HDI modeled the diffusion-weighted signal from each imaging voxel using a combination of anisotropic and isotropic tensor components, as described by Eq (1). Each of the tensor components is described as a standard diffusion tensor formulation in the diagonal coordinate system [27]:

$$S_{k}=\int_{i=1}^{N_{Aniso}}f_{i}e^{-|\vec{b_{k}}|∙\lambda_{⏊\_i}}e^{-|\vec{b_{k}}|∙(\lambda_{\|\_i}-\lambda_{⏊\_i})∙\cos^{2}\psi_{ik}}+\int_{a}^{b}f(D)e^{-|\vec{b_{k}}|D}dD\;(k=1,2,\ldots ,K)$$ [1]

**Fig 1:**
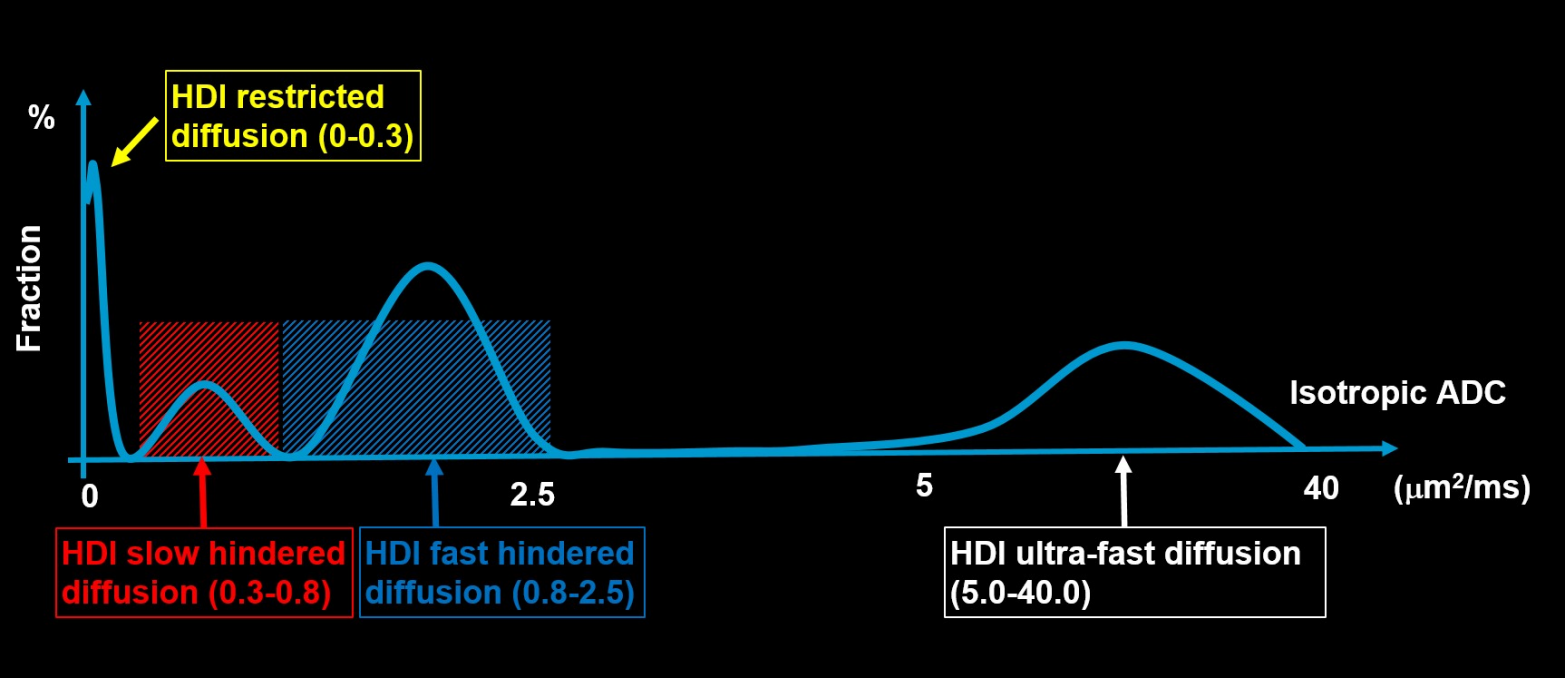
Schematic figure of the isotropic spectrum signals from Heterogeneity Diffusion Imaging. Isotropic diffusivity is used to define each pathological component within a brain tumor. The isotropic diffusivity cutoffs for each of the pathological components were selected from previous diffusion magnetic resonance imaging studies of brain tumors. Specifically, the isotropic diffusion components with diffusivity that ranged between 0.3 and 0.8 μm^2^/ms were associated with the dense packing of tumor cells. The components with diffusivity that ranged between 0.8 and 2.5 μm^2^/ms were associated with extracellular water edema. The components with diffusivity that ranged between 5 and 40 μm^2^/ms were associated with capillary blood perfusion within tumors.

In Eq (1), *|b*_*k*_*|* is the *b* value of the *k*^*th*^ diffusion gradient (*k* = 1, 2, …, K). *S*_*k*_ is the measured diffusion-weighted signal that corresponds with the *k*^*th*^ diffusion gradient. HDI anisotropic components (i.e., the first term on the right side of Eq (1)) were used to model the complex neuronal structures invaded by tumor. *N*_*Aniso*_ is the number of anisotropic tensors in the imaging voxel; *ψ*_*ik*_ is the angle between the *k*^*th*^ diffusion gradient and the principal direction of the *i*^*th*^ anisotropic tensor; *λ*_||_*i*_ and *λ*_*⏊_i*_ are the axial and radial diffusivities, respectively, of the *i*^*th*^ anisotropic tensor; and *f*_*i*_ is the signal intensity fraction for the *i*^*th*^ anisotropic tensor. The HDI isotropic spectrum (i.e., the second term on the right side of Eq (1)) was divided into several nonoverlapping windows on the basis of previously published reports of ADC ranges for different tumor stages [8, 9], and *a* and *b* are the low (0 μm^2^/ms) and high (40 μm^2^/ms) diffusivity limits, respectively, for the isotropic diffusion spectrum *f(D)*. The number of anisotropic and isotropic diffusion components and the signal intensity fractions that correspond with all diffusion components are the key parameters to be solved. The unavoidable measurement and modeling noise will make the direct solution of Eq (1) unstable due to its ill-posed nature [16, 17]. A regularization technique that incorporated the non-negativity of the solution was employed previously in DBSI to stabilize the solution [16, 17], and the same regularization technique was employed in this work. Specifically, isotropic diffusion components with diffusivity between 0 and 0.3 μm^2^/ms were associated with cellularities [16, 17], and those that ranged between 0.3 and 0.8 μm^2^/ms were associated with the slow extracellular diffusion of water trapped between tumor cells, thereby reflecting the packing density of the tumor cells. The isotropic diffusion components with diffusivity that ranged between 0.8 and 2.5 μm^2^/ms were associated with fast extracellular water diffusion, thereby reflecting edema. HDI also acquired diffusion-weighted images with small *b* values to capture the ultrafast isotropic diffusion between 5 and 40 μm^2^/ms. This ultrafast diffusion has been previously described as the intravoxel incoherent motion (IVIM) effect and associated with capillary blood perfusion [28]. Within each imaging voxel for each tumor, the following HDI-derived metrics were quantified: cellularity fraction (CF), slow hindered diffusion fraction (sHF), fast hindered diffusion fraction (fHF), and perfusion fraction (PF). The detailed partition of the isotropic spectrum by HDI is shown in **Fig 1**.

### Surgical treatment

**Fig 2:**
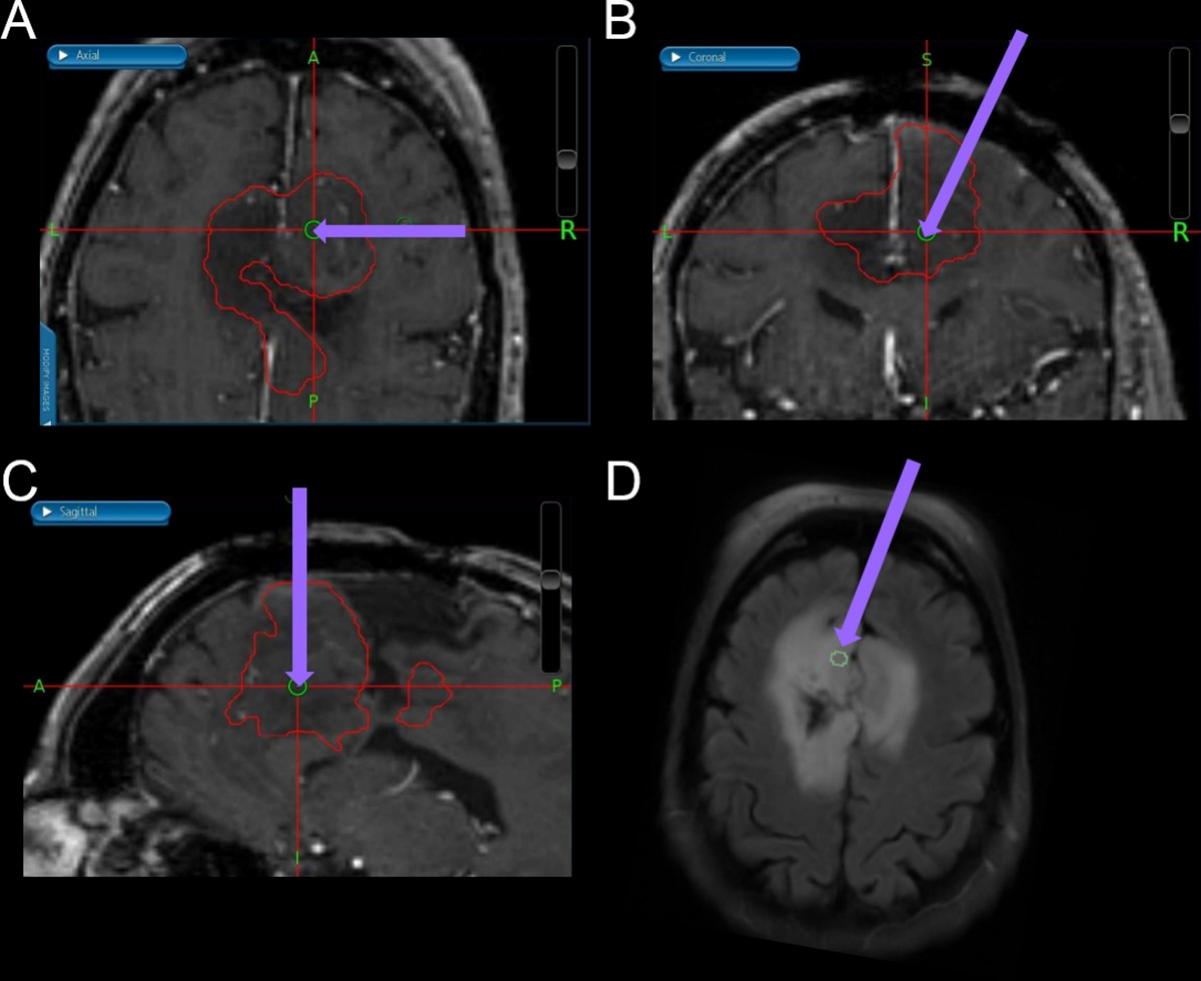
Neuronavigation-guided anatomic images of biopsy tissue sampling sites. Neuronavigation-guided T1-weighted images of the biopsy tissue sampling site at (A) axial, (B) coronal, and (C) sagittal views. (D) The tissue sampling site is labeled on the T2-weighted fluid attentuation inversion recovery image. Purple arrows indicate the passive biopsy needle.

After the MRI, patients underwent stereotactic biopsy followed by partial or complete resection. For each patient, at least one tumor sample was obtained with a stereotactic screen capture taken with the Stealth station. The neuronavigation-guided T_1_W images of the biopsy tissue-sampling sites were shown on axial, coronal, and sagittal views (**Fig 2**). T_1_W contrast-enhanced, T_2_W, and FLAIR images were co-registered by MGA and loaded into the 3D Slicer software platform (http://www.slicer.org) [29] to produce 5-mm diameter spheres that were centered at each surgical tumor sampling site.

### Regions of interest and statistical analysis

Volumetric tumor regions of interest (ROIs) were drawn manually from the FLAIR images. All manual regions of interest (mROIs) were approved by a board-certified neuroradiologist (G.G.). HDI model analysis was performed on the selected mROIs. The ROIs at the tissue sampling sites (tsROIs) were also colocalized on HDI images to quantify the HDI findings. A two-sample *t*-test was performed to compare ADC, CBV, and HDI metrics between the World Health Organization (WHO) II and III tumor groups at tsROIs. The average HDI-derived PF was correlated with the DSC perfusion MRI-derived CBV index. The Pearson correlation coefficient was used to evaluate the association between CBV- and HDI-derived metrics. *P* values of less than .05 were considered significant. Statistical analysis was performed using SAS 9.4 software (SAS Institute Inc., Cary, NC, USA).

## Results

**Fig 3:**
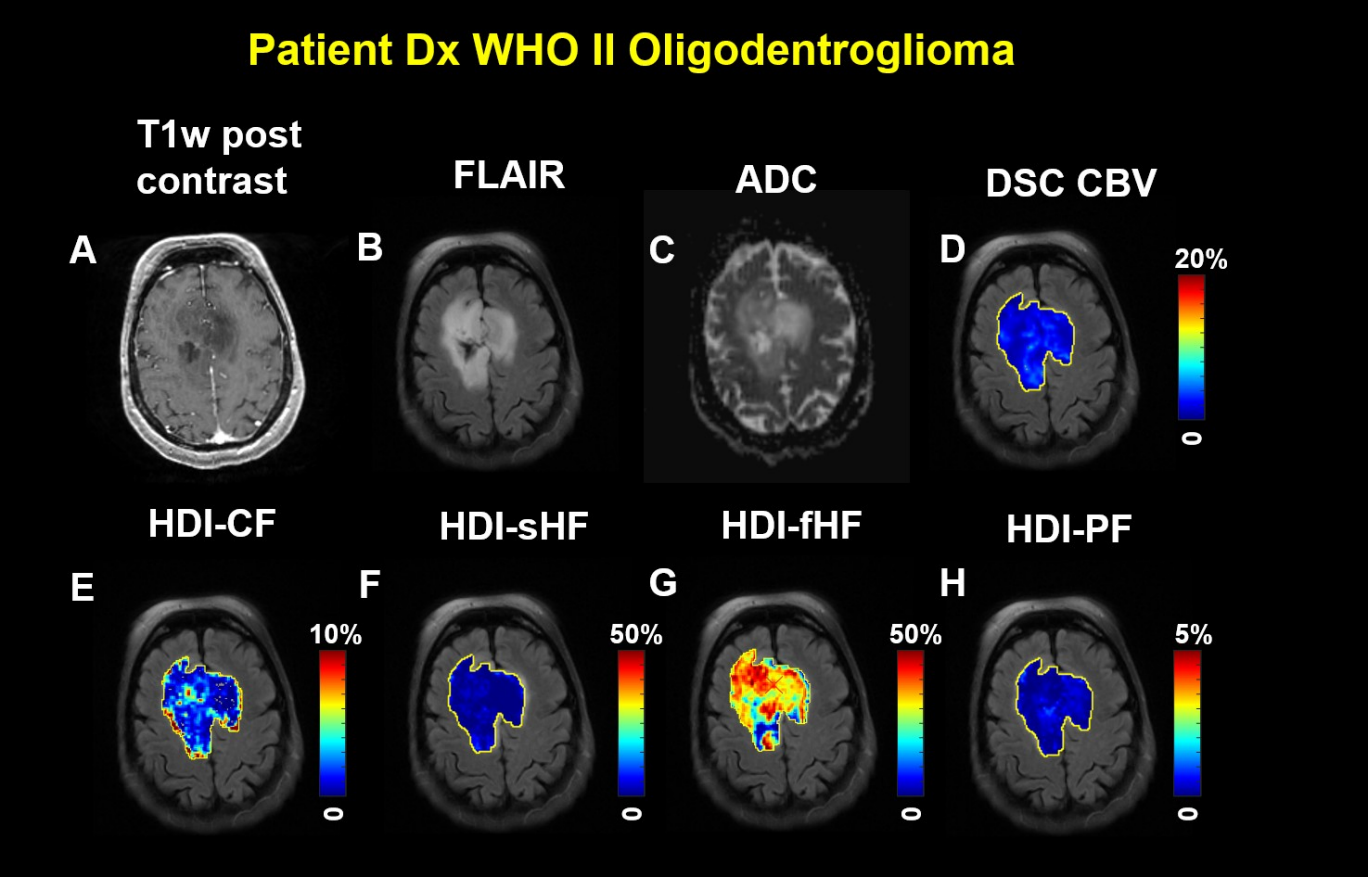
Imaging from a woman in her 70s diagnosed with World Health Organization grade II recurrent oligodendroglioma. (A) The T1-weighted post-contrast image shows a lesion with decreased signal intensity. (B) The fluid-attenuated inversion recovery image and (C) the diffusion magnetic resonance imaging-derived apparent diffusion coefficient show a lesion with an increased signal. (D) The dynamic susceptibility contrast-derived cerebral blood volume map and the Heterogeneity Diffusion Imaging-derived (E) cellularity fraction, (F) slow hindered diffusion fraction, (G) fast hindered diffusion fraction, and (H) perfusion fraction maps were generated on manually defined tumor regions and overlaid on the fluid-attenuated inversion recovery image. No elevated cerebral blood volume and Heterogeneity Diffusion Imaging-derived slow hindered diffusion fraction and perfusion fraction are shown in the tumor region. The elevated Heterogeneity Diffusion Imaging-derived cellularity fraction and fast hindered diffusion fraction are shown in the tumor region.

In all patients, HDI metrics demonstrated heterogeneous spatial distributions within tumors. **Fig 3** shows a representative case from the low-grade tumor group for a woman in her 70s who had been diagnosed with WHO grade II oligodendroglioma. The subject underwent standard-of-care imaging and HDI scanning. The T_1_W post-contrast MRI demonstrated decreased signal intensity in the tumor region (Fig 3A). Elevated FLAIR signal intensity (Fig 3B) and increased ADC (Fig 3C) were also found in the tumor region. Elevated cellularity fraction was found in the HDI-CF map, and the cellularity distribution was heterogeneous within the tumor (Fig 3E). No increased hindered diffusion fraction was identified in the HDI-sHF map (Fig 3F). An elevated hindered diffusion fraction was found in the HDI-fHF map (Fig 3G). A lack of elevated perfusion based on the CBV (Fig 3D) and HDI-PF (Fig 3H) maps was observed in this patient.

**Fig 4:**
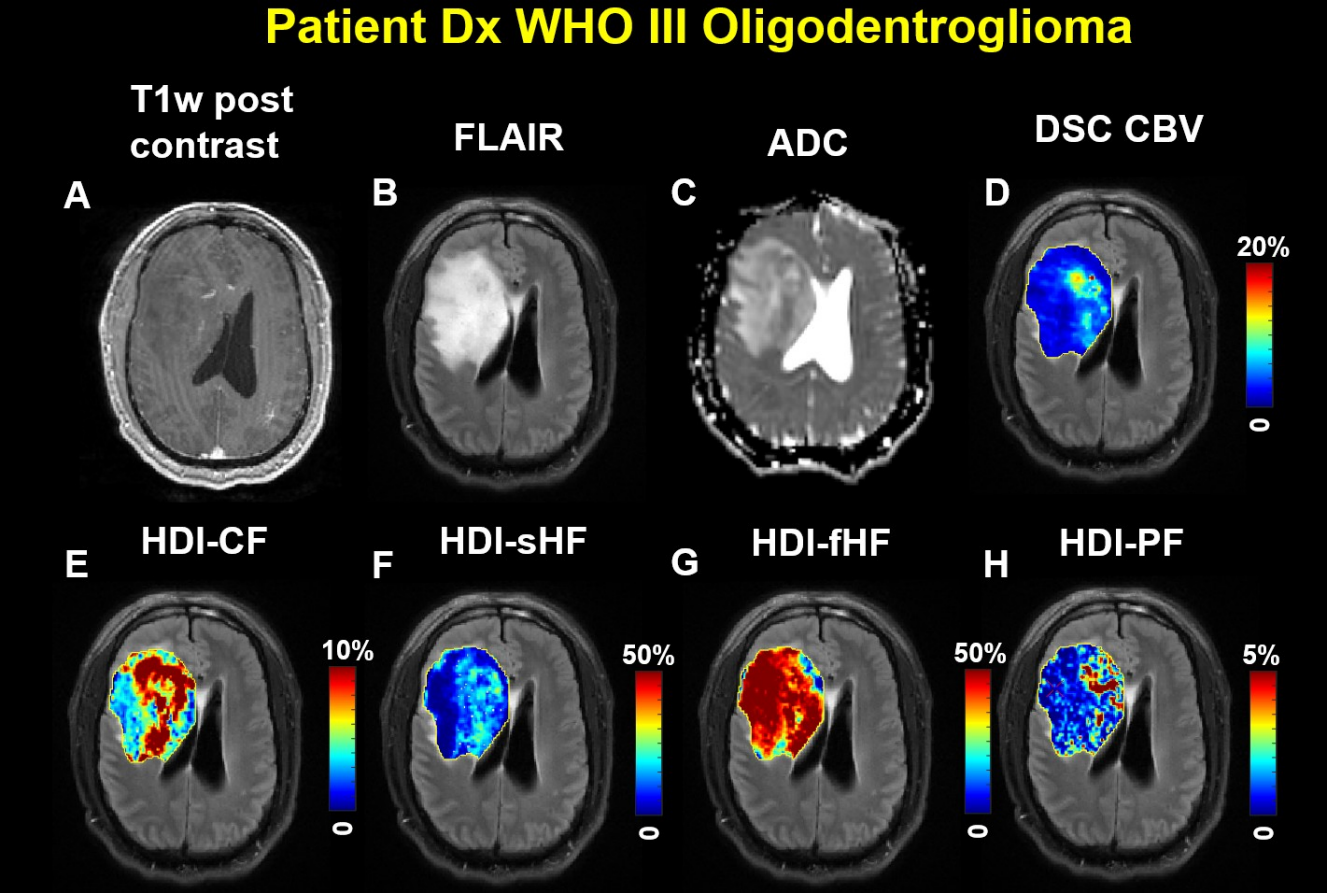
Imaging from a man in his 50s diagnosed with World Health Organization grade III oligodendroglioma. (A) The T1-weighted post-contrast image shows a lesion with decreased signal intensity. (B) The fluid-attenuated inversion recovery image and (C) the diffusion magnetic resonance imaging-derived apparent diffusion coefficient show a lesion with an increased signal. (D) The dynamic susceptibility contrast-derived cerebral blood volume map and the Heterogeneity Diffusion Imaging-derived (E) cellularity fraction, (F) slow hindered diffusion fraction, (G) fast hindered diffusion fraction, and (H) perfusion fraction maps were generated on manually defined tumor regions of interest and overlaid on the fluid-attenuated inversion recovery image. The elevated cerebral blood volume and Heterogeneity Diffusion Imaging-derived cellularity fraction, slow hindered diffusion fraction, fast hindered diffusion fraction, and perfusion fraction are shown in the tumor region.

Another representative case for the high-grade tumor group is shown in **Fig 4** for a man in his 50s who was diagnosed with WHO grade III oligodendroglioma. Representative MRI images of this patient are shown in the figure. T_1_W post-contrast MRI demonstrated decreased signal intensity in the tumor (Fig 4A). Elevated signal intensity (Fig 4B) and increased ADC (Fig 4C) were also found in the tumor. Elevated cellularity fraction was found in the HDI-CF map, and the cellularity distribution was heterogeneous within the tumor (Fig 4E). Elevated sHF (Fig 4F) and fHF (Fig 4G) were observed in this patient. Elevated perfusion based on the CBV (Fig 4D) and HDI-PF (Fig 4H) maps was found in the high-grade brain tumor lesion.

**Fig 5:**
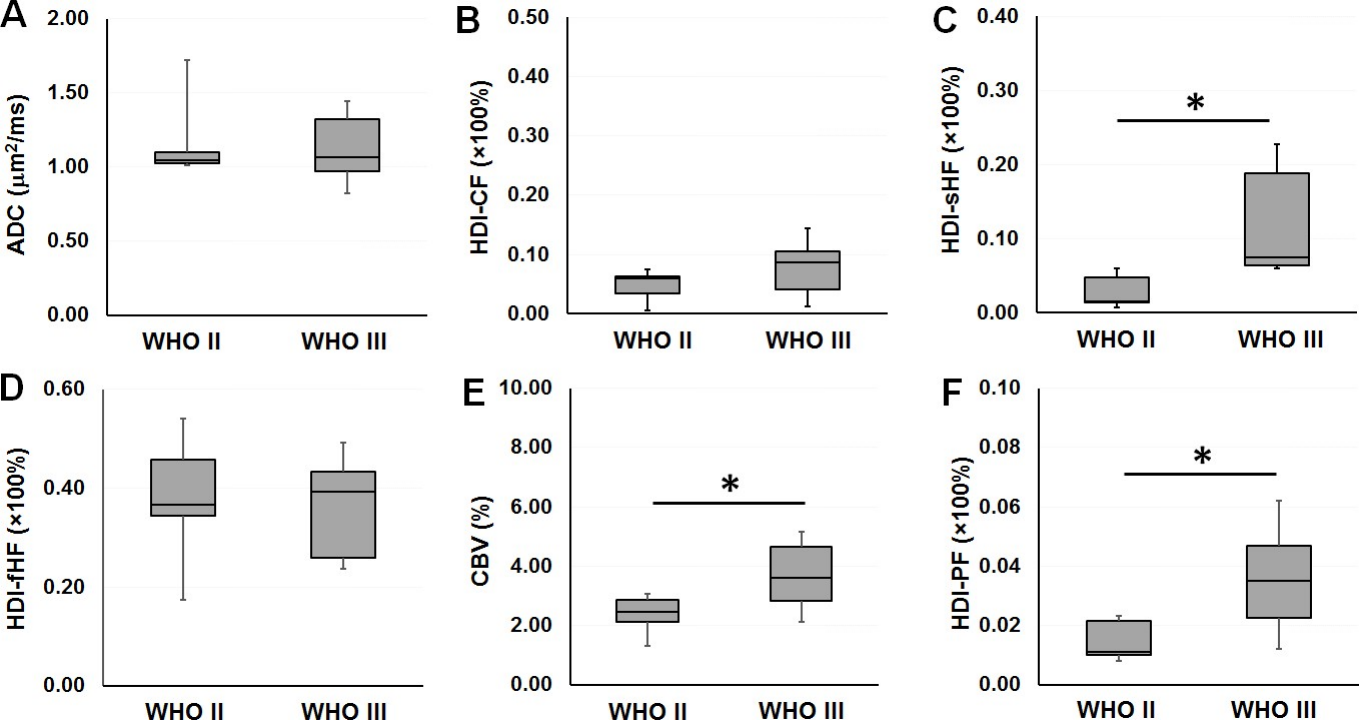
Boxplots of imaging metrics. There is no group significant difference in (A) apparent diffusion coefficient or (B) Heterogeneity Diffusion Imaging (HDI)-derived cellularity fraction between the World Health Organization (WHO) II and III groups. (C) The HDI-derived slow hindered diffusion fraction is significantly higher in the WHO III group as compared with the WHO II group. (D) There is no group significant difference in HDI-derived fast hindered diffusion fraction between the WHO II and III groups. (E) The cerebral blood volume is significantly higher in the WHO III group as compared with the WHO II group. (F) The HDI-derived perfusion fraction is significantly higher in the WHO III group as compared with the WHO II group. Boxes indicate 25^th^ to 75^th^ percentiles, and thin lines indicate 5^th^ and 95^th^ percentiles. *, *P* < .05.

Imaging metrics including ADC, CBV, and HDI-derived indices were compared between the WHO II and III groups at the tissue sampling sites, and details are shown in **Fig 5**. No group significant difference was found in ADC between the WHO II and III groups (Fig 5A). The increasing trend of HDI-derived CF was observed in the WHO III group (Fig 5B). The HDI-derived sHF was significantly elevated in the WHO III group as compared with the WHO II group (Fig 5C). HDI-fHF was not significantly different between the WHO II and III groups (Fig 5D). The CBV generated from DSC perfusion imaging was statistically significantly higher in the WHO III group as compared with the WHO II group (Fig 5E). Similar to the CBV metrics, the HDI-derived perfusion index (PF) was statistically significantly higher in the WHO III group as compared with the WHO II group (Fig 5F).

**Fig 6:**
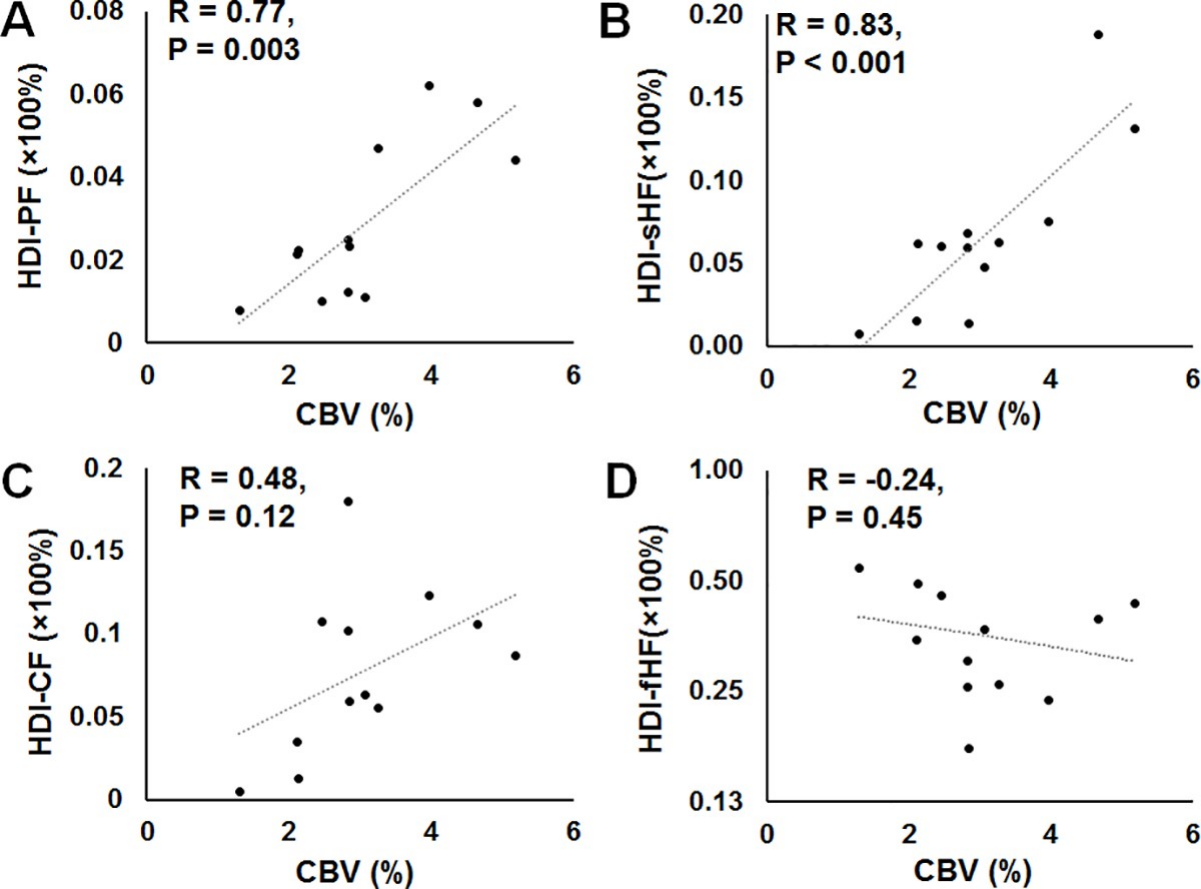
The associations between dynamic susceptibility contrast-derived cerebral blood volume and Heterogeneity Diffusion Imaging-derived indices. Scatter plots showing the significant correlations between (A) dynamic susceptibility contrast perfusion imaging-generated cerebral blood volume (CBV) and Heterogeneity Diffusion Imaging (HDI)-derived perfusion fraction and (B) CBV and HDI-derived slow hindered diffusion fraction in all subjects at the tissue sampling regions. No significant correlations were found between (C) CBV and HDI-derived cellularity fraction or (D) CBV and HDI-derived fast hindered diffusion fraction.

The correlation coefficients between the DSC perfusion imaging and HDI metrics were evaluated, as shown in **Fig 6**. A statistically significant positive correlation was seen between CBV and HDI-derived PF (Pearson’s *r* = 0.77, *P* = .003) (Fig 6A). A significant positive correlation between CBV and HDI-sHF (Fig 6B) was also noted (Pearson’s *r* = 0.83, *P* < .001). CBV was not correlated with HDI-CF or HDI-fHF (Fig 6C and 6D).

## Discussion

Tumor heterogeneity is ubiquitous, especially in malignant tumors [30], which can contain different grades of tumor cells, edema, and vascular structures within each imaging voxel and across the entire tumor. The complexity of the tumor microstructure imposes serious challenges for diagnosis, treatment planning, and post-treatment evaluation. HDI was developed as a novel diffusion MRI technique to characterize tumor heterogeneity by separating and quantifying multiple pathological components.

Although conventional diffusion-weighted imaging (DWI) and DTI have been employed to characterize tumors and to evaluate treatment response in patients with primary brain tumors [9, 12], these methods are not capable of detecting multiple tumor pathologies due to their single diffusion tensor assumption. ADC measures from DWI have been widely used in diagnostic oncology, and the reduced ADC of some tumors has been associated with a high density of tumor cells [9]. However, the sensitivity and specificity of ADC for detecting malignant tumors may be decreased by the edema and/or tumor necrosis that coexists in the lesions [31].

The development of advanced models and model-free diffusion MRI techniques has demonstrated improvements in tumor detection and characterization. For example, by making no assumptions about tissue composition, diffusion kurtosis imaging provides a model-free way to quantify non-Gaussian water diffusion [32]. This type of imaging has demonstrated its sensitivity for grading tumors [33] as well as its high diagnostic accuracy for separating low- from high-grade gliomas through meta-analysis [34]. Similarly, the stretched exponential method—developed to describe diffusion-related signal decay as a continuous distribution, with no assumptions made about the number of participating sources [35]—has demonstrated better performance for differentiating tumor grades as compared with ADC and DTI [36]. Although both the diffusion kurtosis imaging and stretched exponential methods show that non-Gaussian diffusion effects can be used as general heterogeneity biomarkers, it is unknown whether the non-Gaussian diffusion effects are mainly contributed by tumor cells or by the neuronal structures invaded by the tumor. Generalized q-sampling imaging derives complex intravoxel and intervoxel fiber alignment in tissue [37]. A rodent and human glioblastoma study has indicated that q-sampling imaging detected unique intratumor structural features that correlate with both intratumor biological heterogeneity and overall survival [38]. However, no isotropic diffusion components directly associated with tumor cells were included in the present study. Restriction spectrum imaging is an advanced DWI modeling technique that allows for the more direct measurement of tumor cells due to its ability to distinguish among different pools of water within tumor tissues [39]. Previous studies have demonstrated that the restriction spectrum imaging index has increased sensitivity and specificity as compared with ADC for the assessment of brain tumors [39]. Model-based advanced diffusion methods have the advantage of providing more specific subvoxel information. HDI was developed on the basis of the data-driven multicompartment model DBSI [16–20], with the extension of the full isotropic diffusion spectrum to 40 μm^2^/ms. This pilot study demonstrated that HDI is capable of imaging and quantifying multiple tumor pathological components and microvascularity perfusion simultaneously within brain tumors in a single clinical diffusion scan, which distinguishes it from previous models.

In this study, HDI employed CF and sHF images to characterize the spatial distributions of tumor cellularity and its packing density, which cannot be revealed from conventional ADC maps and other advanced diffusion methods. The representative images from one low-grade tumor patient with WHO II oligodendroglioma (see Fig 3) and one high-grade tumor patient with WHO III oligodendroglioma (see Fig 4) showed very heterogeneous tumor cellularity distributions. The conventional ADC measurements were incapable of differentiating between patients with WHO II and III tumors (see Fig 5A), thereby demonstrating ADC’s limited capability for characterizing heterogeneous tumor microenvironments. HDI-CF showed an increased trend in the WHO III group as compared with the WHO II group, but no statistically significant difference was found (see Fig 5B), probably due to the small sample size. Interestingly, HDI demonstrated that the WHO III group had a much higher HDI-sHF than that found in the WHO II group (see Fig 5C), which suggests that there are more densely packed tumor cells in the WHO III group as compared with the WHO II group. The HDI-fHF findings (see Fig 5D) demonstrated that the extracellular water fractions are comparable between the two groups. These findings suggest that HDI parameters can better quantify the microstructural heterogeneity within tumors and that these parameters may provide higher sensitivity for categorizing tumors as compared with conventional ADC. In this pilot study, we demonstrated the feasibility of using HDI to analyze heterogeneous brain tumors. No automatic method was used to classify the tumor grades based on the HDI findings due to the small sample size. A larger study will enable us to better define the relationship between tumor grade and HDI distribution, which will allow for an automatic tumor grade evaluation.

The discovery of gadolinium tissue deposition [40] and the uncertainty surrounding its effects compel the imaging community to find alternative methods for quantifying perfusion. The conventional IVIM method [28] employs a simple biexponential model to quantify perfusion (fast diffusion) and diffusion (slow diffusion) effects in biological tissue. Although conventional IVIM-derived perfusion metrics have improved the diagnostic performance of arterial spin labeling-derived cerebral blood flow and have a strong correlation with cerebral blood flow [41], their accuracy and reliability have not been well accepted, at least partially due to the associated overly simplified biexponential computation model for complex biological tissues. To address this limitation of conventional IVIM, HDI incorporates the “ultrafast” IVIM component associated with capillary blood perfusion [41] into the comprehensive modeling of neuronal components, tumor cellularity, and extracellular water (Eq (1)). In this study, the HDI perfusion component was compared with clinical DSC perfusion imaging in all subjects. The strong correlation between HDI-derived perfusion and DSC-derived CBV (Fig 6A) and the similar spatial distribution patterns between those measures (see Fig 3D and 3H and Fig 4D and 4H) suggest that the HDI-derived perfusion index holds great promise as a complementary noninvasive method for accurately quantifying tumor CBV for patients who cannot receive contrast material for clinical DSC scans. The findings that CBV correlated with HDI-sHF (see Fig 6B) suggest that blood perfusion increases with an increased packing density of tumor cells.

The results of the biopsy evaluation of tumors highly depends on the location of the tissue sampling. The suboptimal selection of the biopsy site may lead to underdiagnosis, undertreatment, and higher tumor recurrence rates. Conventional T_1_W or T_2_W imaging has been previously employed for MRI-guided biopsy [42], and DWI has also been used to optimize biopsy target selection [43]. However, those imaging contrasts may not distinguish tumors from other pathologies such as regions with edema or tumor necrosis. This study has demonstrated that HDI could be a promising noninvasive tool for the guidance of biopsy, surgery, and radiotherapy.

Each biopsy site represents a different region of tumor that is localized by the Stealth neuronavigational system and superimposed on both anatomical maps and maps of HDI-derived indices. Spatial heterogeneity is a fundamental feature of brain tumors [44]. Different sampling regions within one tumor usually have different characterizations, which is evidenced by the diffusion and perfusion data from the same subject (see Fig 4). Thus, including multiple samples from the same subject will increase statistical power without biasing the analysis.

There are several limitations of this pilot study. First, the sample size is small. Future studies with larger numbers of patients will be needed to further validate the HDI technique. Second, the isotropic diffusivity thresholds were selected based on results from previously published DBSI studies. More patient data and histology studies are needed to further refine and optimize the threshold selection. Third, the fixed diffusion time employed in this study could lead to potential overlapping among different isotropic components. Incorporating multiple diffusion time measurement [45] could potentially improve HDI’s accuracy when characterizing the tumor microenvironment.

## Conclusions

HDI was developed in this pilot study to noninvasively characterize brain tumor heterogeneity. The preliminary data demonstrate the capability of HDI to quantify the microenvironment heterogeneity of brain tumors, including tumor cell fraction, packing density, edema, and capillary blood perfusion in a single diffusion MRI examination. The HDI results are consistent with pathology assessments of biopsy tissues and DSC measurements of blood perfusion. Larger studies will be needed to further validate HDI and to establish its role in the clinical management of patients with brain tumors.

## Abbreviations

ADC: Apparent diffusion coefficient
CBV: cerebral blood volume
CF: cellularity fraction
CONDR: Comprehensive Neuro-Oncology Data Repository
DBSI: diffusion basis spectrum imaging
DSC: dynamic susceptibility contrast
DTI: diffusion tensor imaging
DWI: diffusion-weighted imaging
fHF: fast hindered diffusion fraction
FLAIR: fluid-attenuated inversion recovery
HDI: Heterogeneity Diffusion Imaging
IVIM: intravoxel incoherent motion
MGA: Multimodal Glioma Analysis
MRI: magnetic resonance imaging
mROI: manual region of interest
PET: positron emission tomography
PF: perfusion fraction
ROI: region of interest
sHF: slow hindered diffusion fraction
T_1_W: T1-weighted
T_2_W: T2-weighted
TE: echo time
TR: repetition time
tsROI: tissue sampling region of interest
WHO: World Health Organization
